# Prediction of cervical lymph node metastasis from immunostained specimens of tongue cancer using a multilayer perceptron neural network

**DOI:** 10.1002/cam4.5343

**Published:** 2022-10-28

**Authors:** Kohei Kawamura, Chonho Lee, Takashi Yoshikawa, Al‐Shareef Hani, Yu Usami, Satoru Toyosawa, Susumu Tanaka, Shin‐Ichiro Hiraoka

**Affiliations:** ^1^ 1st Department of Oral and Maxillofacial Surgery, Graduate School of Dentistry Osaka University Osaka Japan; ^2^ Cybermedia Center Osaka University Osaka Japan; ^3^ Department of Oral & Maxillofacial Surgery Osaka City University Graduate School of Medicine Osaka Japan; ^4^ Department of Oral Pathology Osaka University Graduate School of Dentistry Osaka Japan

**Keywords:** deep learning, immunohistochemistry, lymphatic metastasis, neural network, tongue neoplasms

## Abstract

**Background:**

Although cervical lymph node metastasis is an important prognostic factor for oral cancer, occult metastases remain undetected even by diagnostic imaging. We developed a learning model to predict lymph node metastasis in resected specimens of tongue cancer by classifying the level of immunohistochemical (IHC) staining for angiogenesis‐ and lymphangiogenesis‐related proteins using a multilayer perceptron neural network (MNN).

**Methods:**

We obtained a dataset of 76 patients with squamous cell carcinoma of the tongue who had undergone primary tumor resection. All 76 specimens were IHC stained for the six types shown above (VEGF‐C, VEGF‐D, NRP1, NRP2, CCR7, and SEMA3E) and 456 slides were prepared. We scored the staining levels visually on all slides. We created virtual slides (4560 images) and the accuracy of the MNN model was verified by comparing it with a hue–saturation (HS) histogram, which quantifies the manually determined visual information.

**Results:**

The accuracy of the training model with the MNN was 98.6%, and when the training image was converted to grayscale, the accuracy decreased to 52.9%. This indicates that our MNN adequately evaluates the level of staining rather than the morphological features of the IHC images. Multivariate analysis revealed that CCR7 staining level and T classification were independent factors associated with the presence of cervical lymph node metastasis in both HS histograms and MNN.

**Conclusion:**

These results suggest that IHC assessment using MNN may be useful for identifying lymph node metastasis in patients with tongue cancer.

## Graphical abstract

Prediction of lymph node metastasis from resected specimens of tongue cancer is clinically useful; MNN can be highly accurate for simple classification with a small amount of training data. This method allows an automatic classification of the staining levels of immunochemically stained specimens, and a correlation was found between CCR7 staining levels and lymph node metastases. This is a potential new system for the assessment of lymph node metastases in patients with oral cancer.

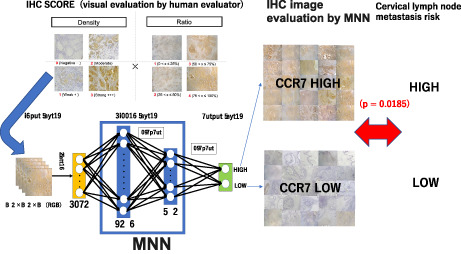

## 1 INTRODUCTION

Oral cancer, a malignant tumor occurring in the oral cavity, is a rare type of cancer with an incidence rate of 1%–5% of all malignant tumors; however, but the cases of oral cancer have increased in recent years. Most of the oral cancer cases are squamous cell carcinomas, with approximately 40% of the cases predominantly affecting the tongue.^1^ Although oral cancer occurs in the area that patients themselves can see, many patients present to specialized medical institutions at a very advanced stage. From 2009 to 2018, tongue cancer mortality rates have reportedly increased by 2% annually.^2^ The survival rates for patients with advanced oral cancer are low, indicating that early detection is most important for survival.^3^ In particular, the presence or absence of cervical lymph node metastasis is an important factor in predicting the therapeutic outcome of oral cancer.^4^ The presence of pathological extranodal extensions of metastatic lymph nodes is associated with poor prognosis and requires additional postoperative treatment with chemoradiation. The diagnosis of cervical lymph node metastasis in patients with oral cancer is made via palpation and imaging techniques, including computed tomography, magnetic resonance imaging, ultrasound echo, and positron emission tomography–computed tomography.^5^ However, the diagnosis by imaging and palpation has limitations, and early cervical lymph node metastases may not be detected.^6^ The recommendation for neck dissection has shifted from radical neck dissection, in which the accessory nerves, sternocleidomastoid muscle, and internal jugular vein are removed, to modified radical neck dissection, in which these tissues are spared to prevent deterioration of the patients' quality of life, such as difficulty in raising the upper arm. However, the National Comprehensive Cancer Network guidelines still recommend elective neck dissection only for cases requiring prophylactic neck dissection.^5^ It also includes a recommendation for sentinel lymph node biopsy.^5^ However, one disadvantage is that sometimes lymph node identification may be technically difficult.

In addition, prophylactic neck dissection is recommended in patients with oral cancer,^7^ but this is still highly debated today.^8^ Although there are many reports on the relationship between the histological and pathological characteristics of the primary tongue tumor and cervical lymph node metastasis,^9^, ^10^, ^11^, ^12^ it is still controversial whether it is a definitive poor prognostic factor.^13^, ^14^

Tumor budding was recently reported as a valid predictor of cervical lymph node metastasis.^15^, ^16^, ^17^, ^18^ In addition, the report has shown that tumors with intratumoral perineural invasion are at a higher risk of cervical lymph node metastasis.^19^ However, it requires advanced diagnostic skills of oral pathologists.

Therefore, predicting the risk of occult cervical lymph node metastasis from the resected specimens of the primary tumor in patients with oral cancer with a clinical diagnosis of N0 would be clinically useful.

We previously reported that immunohistochemical (IHC) staining of resected specimens of tongue cancer for angiogenesis‐ and lymphangiogenesis‐related proteins and visual scoring of the staining levels correlated with lymph node metastasis.^20^ However, this method could not eliminate the visual bias of the evaluator and is time‐intensive because multiple people must visually evaluate the pathological specimens.

McCulloch and Pitts^21^ had conceived a mathematical model that mimicked a neuron. Later, based on this neuron, Rosenblatt et al.^22^ proposed the perceptron, whose parameters can be tuned by learning. By using differentiable functions, such as sigmoid and hyperbolic tangent functions, as activation functions, weights can be propagated, and the model can be learned. By further increasing the layers and complexity, a more flexible model can be built, which is called the multilayer perceptron. In this study, we used a multilayer perceptron neural network (MNN) to evaluate the IHC staining level for angiogenesis‐related proteins and lymph node metastasis in histopathological specimens of tongue cancer. In addition, we compared the accuracy of the hue–saturation–value (HSV) histogram, which visualizes the evaluator's manual interpretation, and verified the accuracy of the MNN automated classification.

## 2 MATERIALS AND METHODS

### 2.1 Patients and tissues

This study enrolled a total of 80 patients who were diagnosed with squamous cell carcinoma of the tongue at the First Department of Oral and Maxillofacial Surgery, Osaka University Dental Hospital, between 2005 and 2015. Among the 80 cases selected, 4 cases were excluded as they were considered inadequate due to the age‐related deterioration of the IHC specimens. Thus, a total of 76 cases were analyzed. The enrolled patients underwent radical resection and were followed up for >5 years to evaluate the presence of lymph node metastasis and prognosis. The patients consisted of 53 males (69.7%) and 23 females (30.3%) ranging in age from 22 to 92 years, with a median age of 62 years. The T classification was based on the eighth edition of the Union for International Cancer Control classification.^23^ Accordingly, 10 patients (13.2%) had T1 stage cancer, 46 (60.5%) had T2 stage cancer, 7 (9.2%) had T3 stage cancer, and 13 (17.1%) had T4 stage cancer. Based on the AJCC 8th staging system, 10 patients (13.2%) had stage 1, 34 patients (44.7%) had stage 2, 8 patients (10.5%) had stage 3, and 24 patients (31.6%) had stage 4 cancer. After >5 years of follow‐up, 37 patients (48.7%) developed pathologic lymph node metastases.

### 2.2 Immunohistochemistry

Paraffin sections were fixed in 10% neutral buffered formalin. Sections of 5‐μm thickness were cut consecutively, deparaffinized in xylene, rehydrated with graded concentrations of ethanol, and treated with citrate buffer at pH 6.0 and 98°C for 30 min followed by EDTA buffer at pH 8.0 and 98°C for 15 min for heat‐induced antigen retrieval. To block endogenous peroxide activity, 0.3% H_2_O_2_·dH_2_O was applied to the sections. Non‐specific reactions were blocked with 1% bovine serum albumin buffer (Histofine SAB‐PO [Multi] kit; Nichirei Bioscience, Tokyo, Japan). The sections were incubated with the following primary antibodies at 4°C overnight: anti‐human rabbit polyclonal VEGF‐C antibody (dilution 1:100), anti‐human rabbit polyclonal VEGF‐D antibody (dilution 1:200) (both from Abcam), anti‐human goat polyclonal CCR7 antibody (dilution 1:250), anti‐human rabbit monoclonal NRP1 antibody (dilution 1:100) (both from Abcam), anti‐human rabbit polyclonal NRP2 antibody (dilution 1:100; Atlas Antibodies), and anti‐human goat polyclonal SEMA3E antibody (dilution 1:100; Abcam). Next, the appropriate secondary antibodies and blocking agents were applied using the Histofine SAB‐PO (Multi or Goat) kit (Nichirei Bioscience). IHC staining and immunolocalization of the proteins were performed using the DAB‐Peroxidase Substrate Solution IHC brown Histofine SAB‐PO (Multi) kit (Nichirei Bioscience), according to the manufacturer's instructions. The sections were counterstained with Mayer's hematoxylin solution (Sigma‐Aldrich), dehydrated with graded concentrations of ethanol, cleared with xylene, and mounted for visualization using bright‐field microscopy. A positive control (IHC staining demonstrating weakly positive tissue) and negative controls (IHC staining omitting the primary antibody) were included in the staining protocol. All 76 specimens were IHC stained for the six types shown above (VEGF‐C, VEGF‐D, NRP1, NRP2, CCR7, and SEMA3E), and 456 slides were prepared. The researchers who performed the staining level scoring and cell counts had no prior clinical knowledge of whether the patients had lymph node metastases.

### 2.3 Data preparation

**FIGURE 1:**
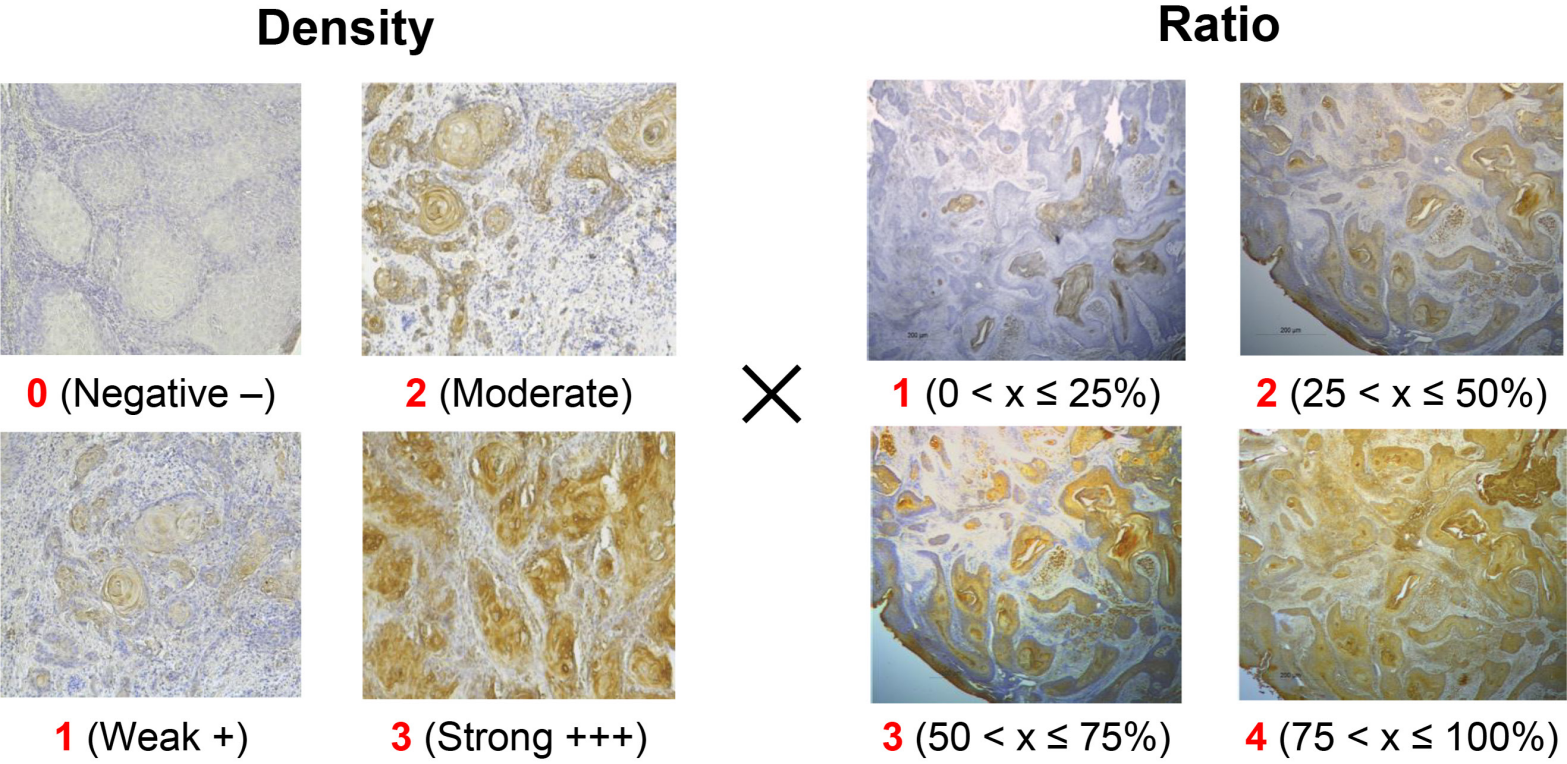
Scoring method for visual evaluation. Density (0–3 points) and ratio (1–4 points) of the hue region immunohistochemical staining

A total of 456 specimens that underwent IHC staining were visually evaluated for density (0–3 points) and ratio (1–4 points) of the stained area by three evaluators specializing in oral surgery and oral pathology, and the product of the two was scored. The results were further confirmed by a supervisor (Figure 1). A virtual slide was created (Virtual Slide System VS120 [OLYMPUS]), the tumor area was confirmed by HE staining, and then the same area of the IHC specimen was photographed to obtain 10 screenshots for each of the 456 slides (a total of 4560 screenshots). Most of the cases had intermediate (3–9) staining level scores, and the scores could vary depending on the researcher's color vision. Some dyed areas, which were difficult to determine, were also included. Therefore, only images that were uniformly stained and that any researcher clearly judged to have a high or low staining level were included in the data set. Altogether, 148 images with a staining level of 10–12 points were designated “high‐staining images,” and 141 images with a staining level of 0–2 points were designated “low‐staining images.” Data augmentation was performed by preprocessing the data using up/down, left/right, 180° rotation, flipping, and zooming to create a dataset of 1008 high and 846 low staining images.

### 2.4 MNN

**FIGURE 2:**
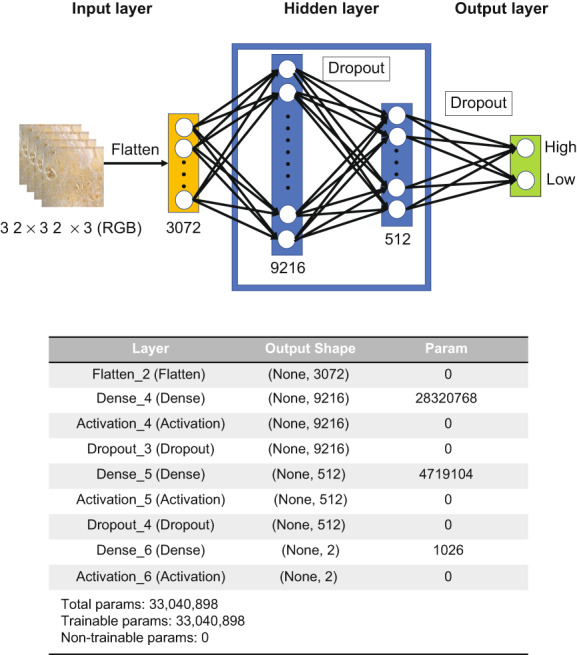
Schematic representation of the multilayer perceptron neural network

A MNN, also known as a forward propagating neural network, is a neural network in which signals propagate in the order of an input layer, an intermediate layer, and an output layer. Except for the input nodes, the individual nodes are neurons that use a nonlinear activation function. MNNs use a supervised learning technique known as error back propagation (back propagation) for training.^24^ MNNs can identify data that is not linearly separable.^25^ In this study, after compressing the image to 32 × 32 pixels and increasing the data six‐fold by augmentation, we used a model that performs two classifications of the high and low staining levels with 9216 and 512 all‐joining layers, respectively (Figure 2). The pathology image data was trained using MNN, and K‐fold cross‐validation (K = 10)^26^ was used to evaluate accuracy. TensorFlow 1.0.8 and Kera 2.0.8 libraries were used for analysis in Python 3.6.

### 2.5 Hue–saturation histogram

**FIGURE 3:**
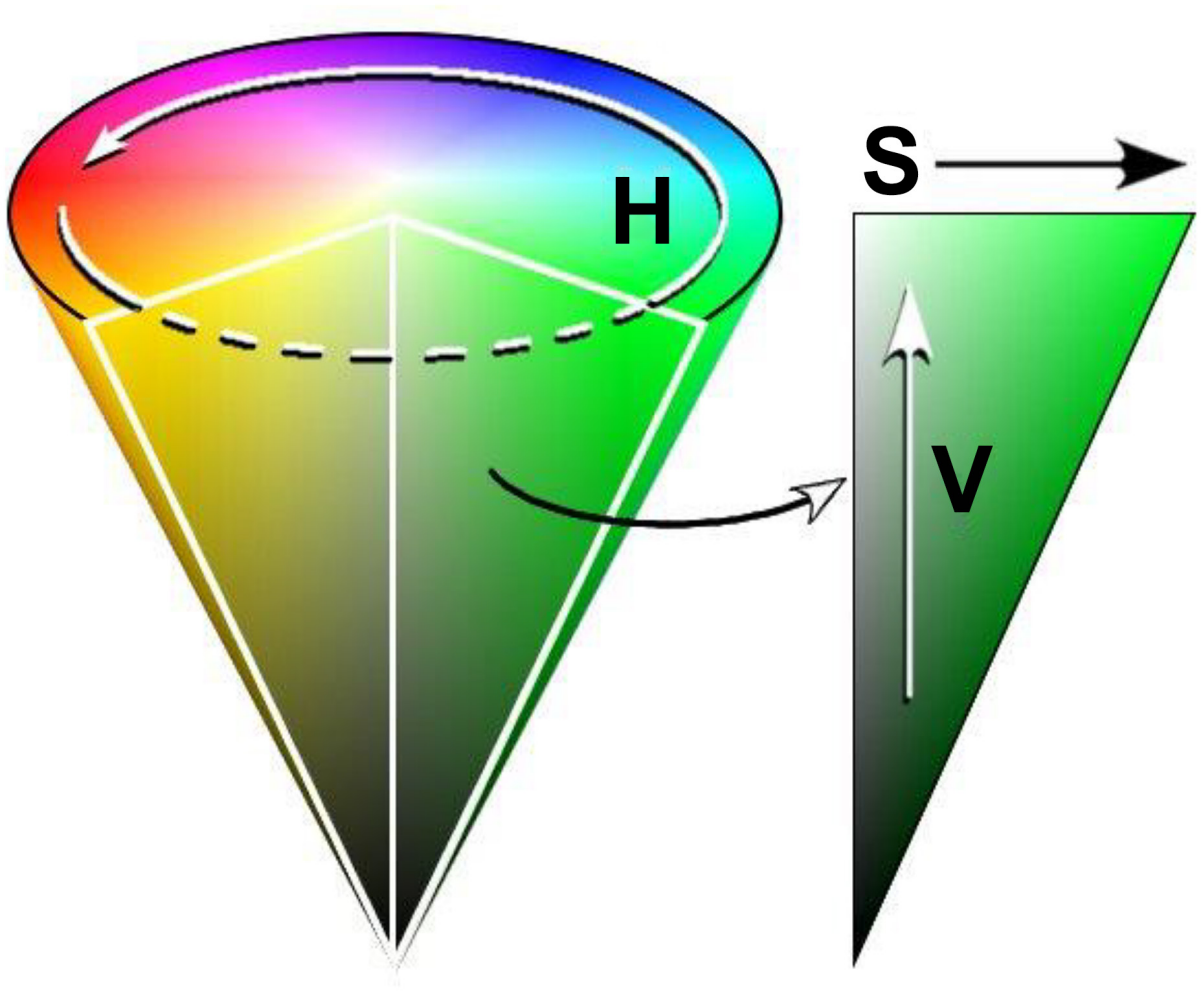
Overview of the HSV color space. A histogram that decomposes an image into three components: H: hue, S: saturation, and V: brightness (“value”). As the brightness V approaches 0, it converges to black regardless of the hue

To verify whether the MNN validation results can evaluate the human visual information correctly, we added a hue–saturation (HS) histogram. The HSV color space (Figure 3)^27^ was devised by Smith et al. with values of 0–255. Hue is displayed as a color bar, and the color level can be quantified. This histogram is a model that approximates human color vision.^28^ The conversion from RGB to HSV is expressed by the following formula:

$$V=\mathrm{MAX}\left(R,G,B\right)$$

$$S=\frac{\mathrm{MAX}\left(R,G,B\right)-\mathrm{MIN}\left(R,G,B\right)}{V}$$

$$H=\cos^{-1}\left\{\frac{\left(G-B\right)+\left(G-R\right)}{2\sqrt{{\left(G-B\right)}^{2}+\left(G-R\right)\left(B-R\right)}}\right\}$$

It has been reported that the brightness (V) is easily affected by the photography conditions and that color image data can be quantified by the HS histogram excluding V.^29^ In this study, the hue (H) regions of high IHC staining were identified, excluding brightness (V), to eliminate the light/dark bias in the imaging and quantified by the saturation (S) level and number of pixels in these regions.

**FIGURE 4:**
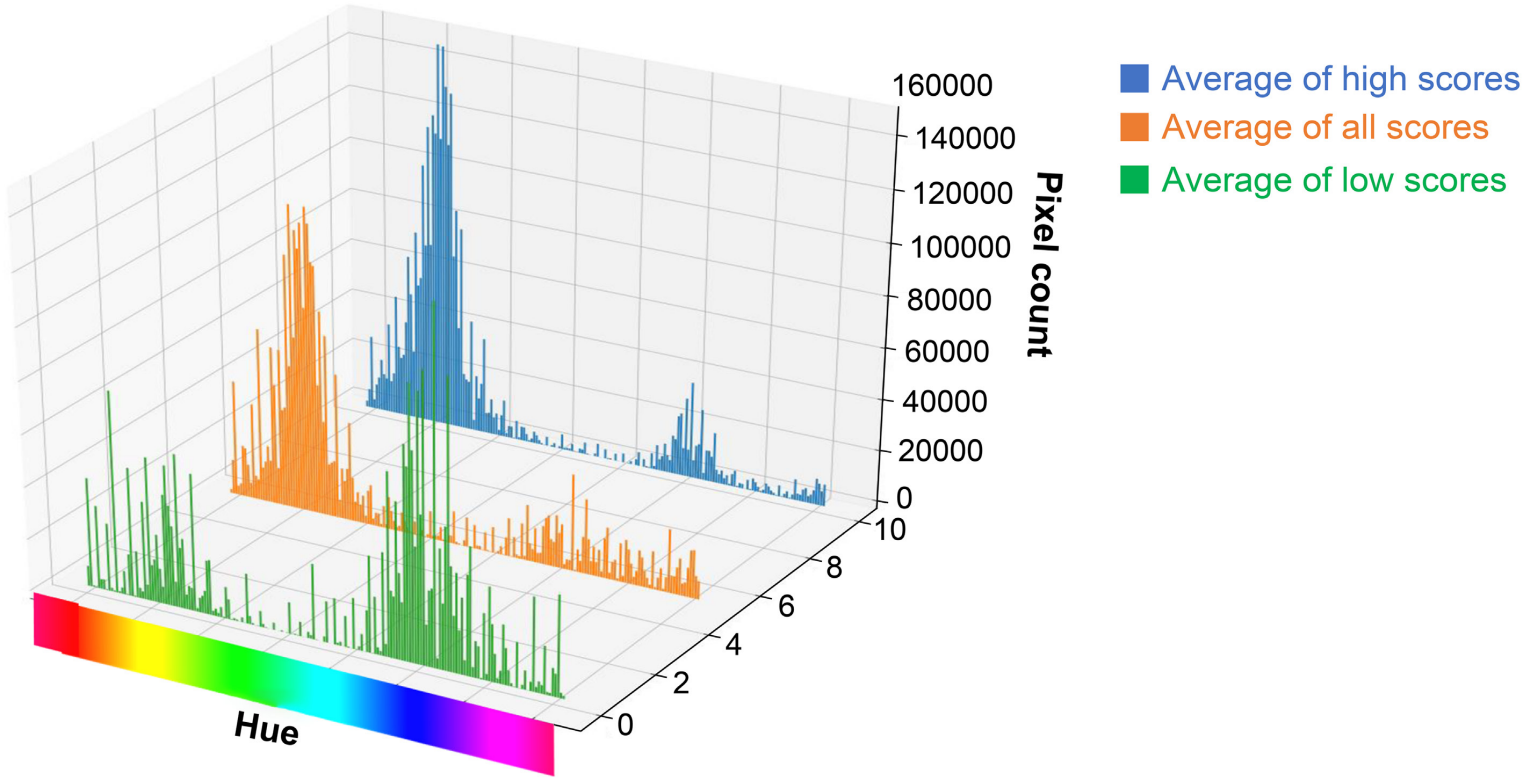
Three‐dimensional graph of the average number of pixels corresponding to the hue for the high and low staining levels and across all images

**FIGURE 5:**
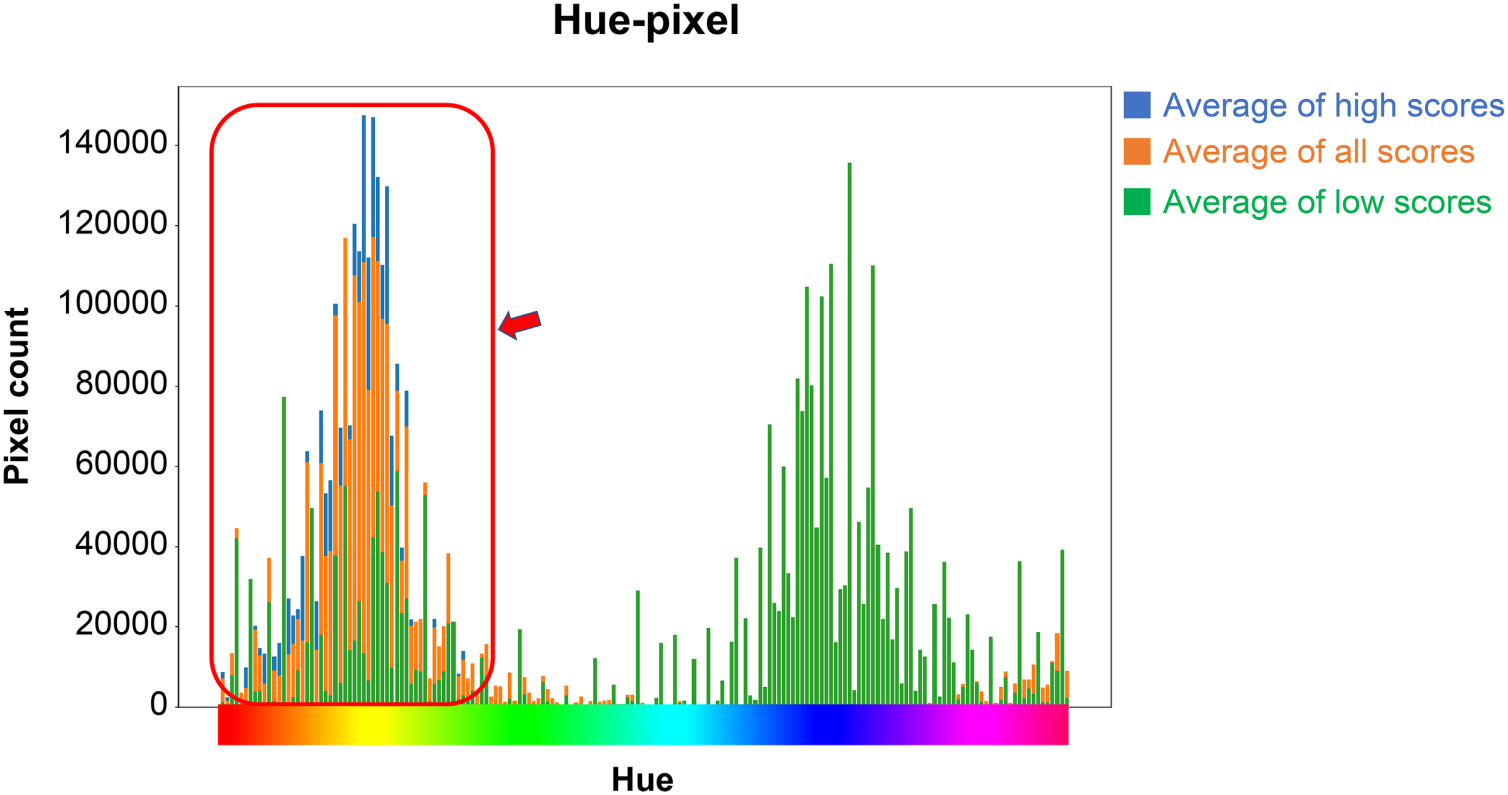
Superimposed graph of Figure 5. The area with the high average > the average of all images > the low average is the area where IHC staining is positive

**FIGURE 6:**
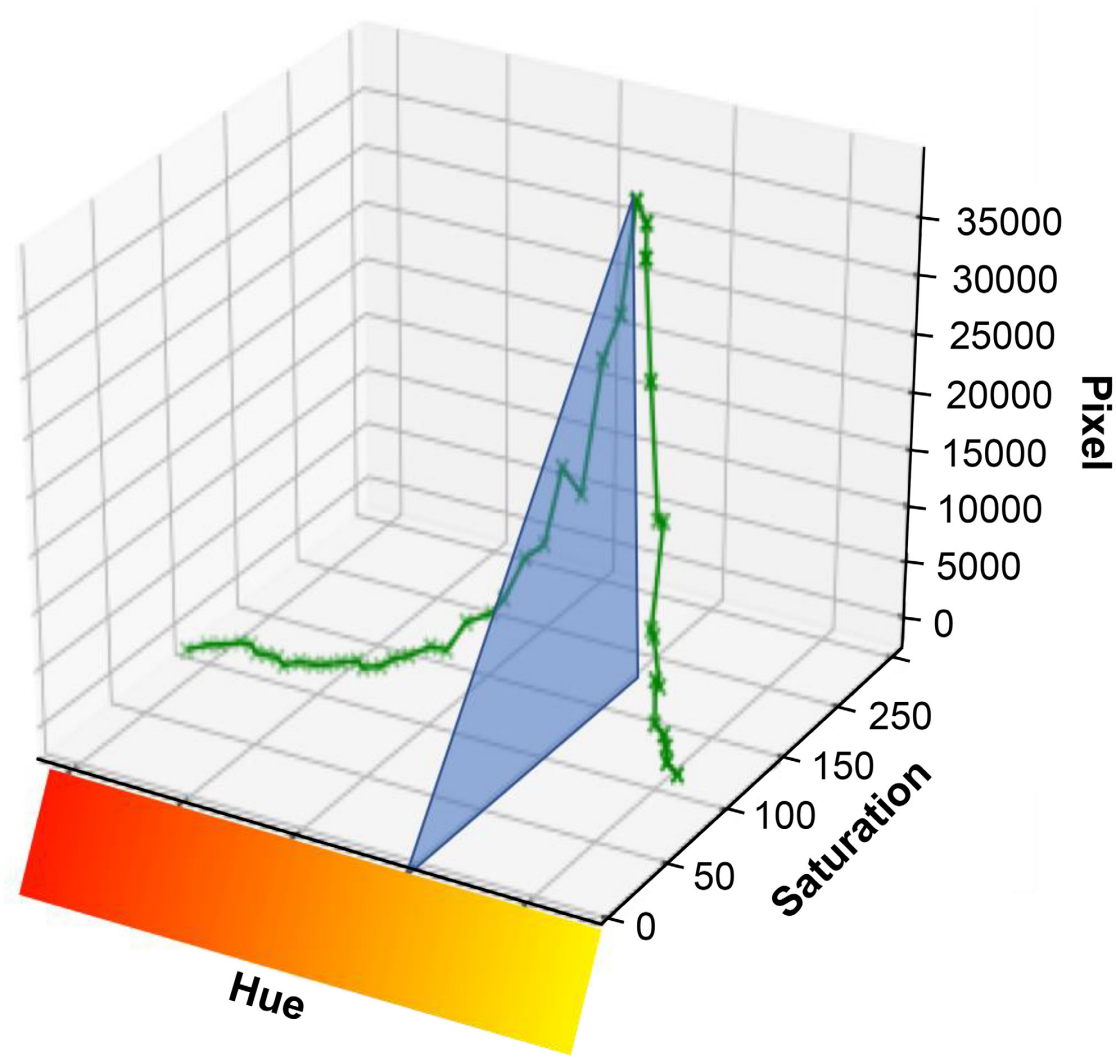
Three‐dimensional graphical quantification of hue (H), saturation (S), and pixels of staining

Among the visual scoring data of the IHC images, the highest staining level score was set to 12, and the lowest score was set to 0. A histogram of the average number of pixels corresponding to the hue of the image is shown in Figure 4. These histograms were superimposed (Figure 5), and the hue region with the average of high staining > the average of all images > the average of low staining was the region used for visual evaluation. A three‐dimensional graph (Figure 6) of the saturation and the number of pixels for the hue area was drawn for all the image data, and quantification was calculated by summing the areas using the following formula:

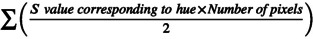

The results were classified by histogram analysis, with values higher than the median value obtained from the results considered to be high and lower values as low.

### 2.6 Statistical analysis

Chi‐square test and logistic regression analysis were performed to assess the correlation between high and low cases and the presence of cervical lymph node metastasis, respectively.

*p* < 0.05 was defined as a statistically significant difference. Statistical analysis was performed with IBM SPSS Statistics v.21 (IBM).

## 3 RESULTS

### 3.1 Accuracy of the MNN learning model

The accuracy of the training model by MNN was 98.6%. To confirm that the model was not affected by morphological features rather than the hue and saturation, we converted the training image to grayscale and trained it under the same conditions, and the accuracy decreased to 52.9%. This result indicated that our MNN adequately assessed the staining levels rather than the morphological features of IHC images.

### 3.2 Automatic classification by MNN

We performed statistical analysis on the results of the automatic classification of the IHC image data into high and low groups by MNN and the association results with the presence of cervical lymph node metastasis (Table 1). We found that the CCR7 staining level (*p* = 0.0165) and T classification ([T1 + T2] / [T3 + T4]; *p* = 0.0499) in the univariate analysis (Chi‐square test) and the CCR7 staining level (*p* = 0.011) and T classification (*p* = 0.034) in the multivariate analysis (logistic regression; Table 2) were associated with the presence of cervical lymph node metastasis.

**TABLE 1:** Results of classification by MNN. The results of the automatic classification of IHC specimens into high and low groups based on MNN and the number of patients in these groups with cervical lymph node metastasis (N+) and without (N−)

	N+	N−
NRP1		
High	28	26
Low	9	13
NRP2		
High	29	22
Low	8	17
CCR7		
High	28	18
Low	9	21
VEGFC		
High	29	23
Low	8	16
VEGFD		
High	22	21
Low	15	18
SEMA3E		
High	33	27
Low	4	12

**TABLE 2:** Association of risk factors with cervical lymph node metastasis based on the results of the automatic classification with MNN. The risk factor is cervical lymph node metastasis

Chi‐square test	Logistic regression analysis					
	Univariate analysis			Multivariate analysis		
	Odds	95% CI	*p*‐value	Odds	95% CI	*p*‐value
NRP1	1.556	0.570–4.243	0.5402			
NRP2	2.801	1.023–7.664	0.0730			
CCR7	3.630	1.362–9.671	0.0165	3.738	1.349–10.360	0.011
VEGFC	2.522	0.919–6.923	0.1159			
VEGFD	1.257	0.506–3.121	0.7933			
SEMA3E	3.667	1.060–12.679	0.0641			
(T1 + T2) / (T3 + T4)	3.348	1.120–10.003	0.0499	1.865	1.048–3.319	0.034

Abbreviation: CI, confidence interval.

### 3.3 Classification by HS histogram

Statistical analysis was performed on the results of sorting into high and low groups according to the median of the HS histogram analysis and the association results with the presence of cervical lymph node metastasis (Table 3). Multivariate analysis showed that the CCR7 staining level (*p* = 0.0185) and T classification (*p* = 0.0328) were independent factors associated with the presence of cervical lymph node metastasis (Table 4).

**TABLE 3:** Results of classification by HS histogram. The results of the automatic classification of IHC specimens into high and low groups based on HS histogram and the number of patients in the group with cervical lymph node metastasis (N+) and without (N−)

	N+	N−
NRP1		
High	22	20
Low	15	19
NRP2		
High	24	19
Low	13	20
CCR7		
High	22	10
Low	15	29
VEGFC		
High	19	15
Low	18	24
VEGFD		
High	12	8
Low	25	31
SEMA3E		
High	32	25
Low	5	14

**TABLE 4:** Association of risk factors with cervical lymph node metastasis based on the results of the HS histogram analysis. Risk factor is cervical lymph node metastasis

Chi‐square test	Logistic regression analysis					
	Univariate analysis			Multivariate analysis		
	Odds	95% CI	*p*‐value	Odds	95% CI	*p*‐value
NRP1	1.393	0.562–3.456	0.6271			
NRP2	1.943	0.773–4.885	0.2348			
CCR7	4.253	1.607–11.257	0.0059	3.625	1.241–10.586	0.0185
VEGFC	1.689	0.678–4.204	0.3696			
VEGFD	1.86	0.659–5.253	0.3581			
SEMA3E	3.667	1.060–12.679	0.0469	2.188	0.603–7.931	0.2333
(T1 + T2) / (T3 + T4)	3.348	1.120–10.003	0.0499	1.897	1.054–3.416	0.0328

Abbreviation: CI, confidence interval.

The validation analysis of MNN and the HS histogram showed comparable results. This suggests that the automatic classification of the learning model by MNN can identify cervical lymph node metastasis from primary tongue tumors.

## 4 DISCUSSION

Although many studies have investigated the pathological and histological features of poor prognosis related to resected oral cancer specimens and the risk of lymph node metastasis,^14^, ^15^, ^16^, ^17^, ^18^, ^19^ imaging and palpation are commonly used clinically to diagnose the presence of lymph node metastasis. However, the diagnosis by imaging also has detection limitations; therefore, it is difficult to detect all occult metastases.

In general, deep learning (DL), represented by convolutional neural networks, is used in the development of medical artificial intelligence, which has advanced rapidly in recent years. The more complex a neural network is, the more data are needed to improve the accuracy of DL training. However, unless a large amount of data can be acquired in daily clinical practice, such as chest radiographs, there are limitations on data collection at a single facility. On the other hand, some neural networks, such as the MNN used in this study, are expected to improve accuracy even if the amount of training data is not large, as long as they perform simple classification.

The results of this study show that MNN can classify features of color information of digitized IHC specimens to create a learning model that can evaluate objective staining levels. Furthermore, this MNN learning model showed that the level of CCR7 staining could be useful for the objective prognostic evaluation of lymph node metastasis. Visual evaluation depends on the evaluator's color vision and their attention to points of interest, and intermediate scores near the boundary between high and low staining could be misjudged by the evaluator. On the other hand, our DL model, which learned the weights associated with specific features, can evaluate specimens with intermediate scores with greater accuracy. Therefore, we believe that the model constructed in this study can be used to assess the level of staining with less bias.

The chemokine receptor, CCR7, is regulated by two endogenous ligands, CCL19 and CCL21.^30^ Its activation plays an important role in the effective control of various immune responses.^31^ Further, various cancer types overexpressing CCR7 have been reported to have a poor prognosis owing to accelerated lymph node metastasis.^32^, ^33^, ^34^, ^35^, ^36^, ^37^ A clinical trial of a small molecule antagonist of CCR7 inhibiting lymph node metastasis is currently underway.^38^ Wang et al. reported that CCL21 stimulation of CCR7 may upregulate the expression of MUC1, a transmembrane glycoprotein of the mucin family, and promote cervical lymph node metastasis in tongue cancer.^39^ These reports suggest the relevance of the results of our study. Based on these results, we plan to further develop prognostic methods for oral cancer in the future.

The current study had several limitations. First, the number of cases was limited due to the single‐center design of this study. Therefore, during the clinical application of DL techniques, it is also necessary to solve problems that cause the performance degradation of machine learning models, such as overfitting,^40^ wherein unknown data cannot fit, and domain shift,^41^ wherein the distribution of training and test data do not match. In the future, it will be necessary to normalize the differences between the centers, such as staining intensity, when preparing immunostained specimens. Faryn et al. reported that using the RandAugment framework as a data augmentation method for HE‐stained images improved its versatility.^42^ Evidence has demonstrated that image normalization using Cycle‐GAN can be effective after adding pathology images from multiple centers,^43^ Future prospective studies using multicenter images are required.

Recently, various studies have been conducted to address the problem of domain shift in medical images. Domain adaptation (DA), fine‐tuning, and federated learning^44^, ^45^, ^46^ are effective solutions to the domain shift in our study.

DA is a form of transfer learning that transfers knowledge across domains by learning invariant features that align the distribution of domains. DA assumes that the original data used for training are labeled, and depending on the target domain, they can be either (1) supervised DA (all labeled datasets), (2) semisupervised DA (few labeled datasets), or (3)unsupervised DA (all unlabeled datasets). The domain of interest envisioned in our MNN model for evaluating staining levels in IHC images is semisupervised or unsupervised DA, given that it is an evaluation of staining levels that does not require specialized pathological knowledge and is relatively easy to label. Although unsupervised DA is considered to be the most difficult, Ganin et al. reported that incorporating a gradient reversal layer improves the accuracy of the model, and we believe that adaptation is possible for our model as well.^47^

Furthermore, Takahashi et al. reported that fine‐tuning of learned models in glioma segmentation from brain MRI can solve the domain shift problem even when the number of cases is <20 at other institutions.^43^ Since the IHC images we used are simpler data than brain MR images, we consider that it is practical to overcome the domain shift problem.

In addition, if we conduct joint research at other institutions and collect sufficient pathology data at each institution, we can solve the domain shift problem by training our MNN algorithm on data from each institution and integrating the parameters using federated learning, without exchanging clinical data.^44^ However, since the images we used were completely anonymized, we believe that they can be collected without the need for such anonymization.

Since all of these methods are based on the assumption that data from different domains are available, this is an issue that should be investigated in future collaborative studies at other institutions. The learning model developed demonstrated the usefulness of CCR7 immunohistochemistry for the diagnosis of cervical lymph node metastasis in tongue cancer. However, for clinical application, comparing the accuracy of the developed learning model with that of human medical practitioners (experts, mid‐levels, and trainees) is needed. In the future, we plan to develop a new system to evaluate the need for prophylactic neck dissection in patients with oral cancer.

This study showed that an MNN learning model could automatically classify the staining level of IHC stained specimens of resected tissue of tongue cancer. Additionally, we found that there was a correlation between the staining level of CCR7 and lymph node metastasis in tongue cancer samples. Therefore, this study suggests that the evaluation of CCR7 staining level by MNN may be useful in diagnosing the presence of cervical lymph node metastasis in tongue cancer.

## AUTHOR CONTRIBUTIONS

KK: Research, formal analysis, writing–draft; CL and TY: Software, resources, visualization; AH, YU, and ST: Evaluation of histopathological specimens; ST: Methodology; SH: Concept building, methodology, validation, formal analysis, research, resources, evaluation of histopathological specimens, data curation, writing–review, supervision, project management.

## FUNDING INFORMATION

This work was supported by JSPS KAKENHI Grant Numbers 20K10115 and 21K17113.

## CONFLICT OF INTEREST

The authors declare no competing interests. The funders had no role in the design of the study; in the collection, analyses, or interpretation of data; in the writing of the manuscript; or in the decision to publish the results.

## ETHICS APPROVAL

The study was conducted in accordance with the Declaration of Helsinki and was approved by the ethical committee of the Osaka University Dental Hospital (approval no. H29‐E35).

## PATIENT CONSENT STATEMENT

Opt‐out consent was obtained for this study.

## PERMISSION TO REPRODUCE MATERIAL FROM OTHER SOURCES

Not applicable.

## CLINICAL TRIAL REGISTRATION

Not applicable.

## Data Availability

The datasets generated and/or analyzed in this study have not been published owing to ethical concerns regarding patient confidentiality but are available from the corresponding authors upon reasonable request.
